# 

**Published:** 1923-07

**Authors:** 


CONTENTS.

*Page*

The Long Fox Lecture: The Relation between Chemistry and Medicine. By Professor F. Francis, D.Sc. ... ... ... ... 119

Injuries to the Head Illustrating Functions of the Cortex. By R. G. Gordon, M.D., M.R.C.P. (Edin.) ... ... ... ... 139

Hoarseness: The Importance of Early Laryngoscopy. By E. Watson-Williams, M.C., B.A., M.B. (Cantab.), Ch.M. (Bristol), F.R.C.S.E. ... ... ... ... ... ... ... 153

Reviews of Books ... ... ... ... ... ... ... ... ... 157

Editorial Notes ... ... ... ... ... ... ... ... ... 167

Meetings of Societies ... ... ... ... ... ... ... ... 170

Local Medical Notes ... ... ... ... ... ... ... ... 174

